# Mechanisms of Cardiorenal Protection With SGLT2 Inhibitors in Patients With T2DM Based on Network Pharmacology

**DOI:** 10.3389/fcvm.2022.857952

**Published:** 2022-05-23

**Authors:** Anzhu Wang, Zhendong Li, Sun Zhuo, Feng Gao, Hongwei Zhang, Zhibo Zhang, Gaocan Ren, Xiaochang Ma

**Affiliations:** ^1^Xiyuan Hospital, China Academy of Chinese Medical Sciences, Beijing, China; ^2^Graduate School, China Academy of Chinese Medical Sciences, Beijing, China; ^3^Qingdao West Coast New Area People's Hospital, Qingdao, China; ^4^Beijing University of Chinese Medicine, Beijing, China; ^5^Clinical Research Center for Chinese Medicine Cardiology, Beijing, China

**Keywords:** type 2 diabetes mellitus, chronic kidney disease, heart failure, sodium-glucose cotransporter 2 inhibitors, cardiorenal protection

## Abstract

**Purpose:**

Sodium-glucose cotransporter 2 (SGLT2) inhibitors have cardiorenal protective effects regardless of whether they are combined with type 2 diabetes mellitus, but their specific pharmacological mechanisms remain undetermined.

**Materials and Methods:**

We used databases to obtain information on the disease targets of “Chronic Kidney Disease,” “Heart Failure,” and “Type 2 Diabetes Mellitus” as well as the targets of SGLT2 inhibitors. After screening the common targets, we used Cytoscape 3.8.2 software to construct SGLT2 inhibitors' regulatory network and protein-protein interaction network. The clusterProfiler R package was used to perform gene ontology functional analysis and Kyoto encyclopedia of genes and genomes pathway enrichment analyses on the target genes. Molecular docking was utilized to verify the relationship between SGLT2 inhibitors and core targets.

**Results:**

Seven different SGLT2 inhibitors were found to have cardiorenal protective effects on 146 targets. The main mechanisms of action may be associated with lipid and atherosclerosis, MAPK signaling pathway, Rap1 signaling pathway, endocrine resistance, fluid shear stress, atherosclerosis, TNF signaling pathway, relaxin signaling pathway, neurotrophin signaling pathway, and AGEs-RAGE signaling pathway in diabetic complications were related. Docking of SGLT2 inhibitors with key targets such as GAPDH, MAPK3, MMP9, MAPK1, and NRAS revealed that these compounds bind to proteins spontaneously.

**Conclusion:**

Based on pharmacological networks, this study elucidates the potential mechanisms of action of SGLT2 inhibitors from a systemic and holistic perspective. These key targets and pathways will provide new ideas for future studies on the pharmacological mechanisms of cardiorenal protection by SGLT2 inhibitors.

## Introduction

Diabetes mellitus (DM) is one of the world's most common public health issues, with the 10th edition of the International Diabetes Federation estimating a prevalence of 537 million people in 2021, and ~783.2 million people are expected to have DM by 2045 (1). Type 2 Diabetes Mellitus (T2DM) is the most common type of diabetes, accounting for more than 90% of all diabetic patients and being one of the primary causes of chronic kidney disease (CKD) and cardiovascular disease (CVD) (2). For instance, in clinical trials for patients with T2DM, the prevalence of heart failure (HF) at baseline ranged from ~10 to 30% (3–5). In clinical trials of patients with chronic HF, the prevalence of T2DM was nearly 30% regardless of HF phenotype (6–8). T2DM is found in ~40–45% of patients hospitalized with HF in North America and Europe (9, 10).10% of deaths in patients with T2DM are attributed to renal failure (11). According to the China Kidney Disease Network 2015 Annual Data Report, the prevalence of CKD is 10.8%, and diabetic nephropathy accounting for 26.9% of CKD hospitalizations (12). Diabetic macrovascular and microangiopathy result in cardiovascular and renal endpoint events, which are the leading cause of death in diabetic patients. In contrast, the improved mortality outcomes obtained with specific classes of hypoglycemic agents are largely independent of their glycemic effects (13).

Sodium-glucose cotransporter 2 (SGLT2) is a key glucose transporter protein in the kidney that accounts for nearly 90% of glucose reabsorption from primary urine, and SGLT2 inhibitors are novel oral drugs for the treatment of T2DM. The United States Food and Drug Administration and the European Union have currently approved four SGLT2 inhibitors (Canagliflozin, Empagliflozin, Dapagliflozin, and Ertugliflozin) (14). Ipragliflozin, Tofogliflozin, and Luseogliflozin are some of the other medications in the family that have been authorized in Japan (15–17). Recent evidence suggests that SGLT2 inhibitors can help individuals with or without T2DM improve their renal and cardiovascular outcomes (Table 1).

**Table 1:** Large clinical trials of SGLT2 inhibitors.

**Drug**	**Trial**	**Patients**	**Median observation time**	**Primary outcome, [HR (95% CI), *P*-value]**
Empagliflozin	EMPA-REG (18)	7,020 T2DM patients with CVD and Egfr ≥30 mL/min/1.73 m^2^	3.1 years	MACE^a^,0.86 (0.74–0.99), *P =* 0.04
	EMPEROR-Reduced (19)	3,730 patients with HFrEF	16 months	CVD death or hospitalization for HF, 0.75 (0.65–0.86), *P* < 0.001
	EMPEROR-Preserved (20)	5,988 HF patients with EF > 40%	26.2 months	CVD death or hospitalization for HF, 0.79 (0.69–0.90), *P* < 0.001
Canagliflozin	CREDENCE (21)	4,401 patients with T2DM and CKD^b^	2.62 years	ESKD^c^, a doubling of the serum creatinine level, or death from renal or CVD, 0.70 (0.59–0.82), *P* < 0.001
	CANVAS (22)	10,142 T2DM patients with CVD risk factors, eGFR≥30 mL/min/1.73 m^2^	126.1 weeks	MACE^d^,0.86 (0.75–0.97), *P =* 0.02
Dapagliflozin	DECLARE-TIMI (23)	17,160 T2DM patients with ASCVD or CVD risk factor	4.2 years	MACE^e^,0.93 (0.84–1.03), *P =* 0.17
	DAPA-HF (24)	4,744 patients with HFrEF, NT-proBNP≥600 pg/mL	18.2 months	Worsening HF^f^ or CVD death, 0.74 (0.65–0.85), *P* < 0.001
	DAPA-CKD (25)	4,304 patients with eGFR 25 to 75 mL/min/1.73 m^2^and UACR 200 to 5,000 mg/g	2.4 years	A sustained decline in the eGFR of at least 50%, ESKD^g^, or death from renal or CVD causes, 0.61 (0.51–0.72), *P* < 0.001
Ertugliflozin	VERTIS-CV (26)	8,246 T2DM patients with ASCVD and eGFR≥30 mL/min/1.73 m^2^	3.5 years	MACE^h^, 0.97 (0.85–1.11), *P* < 0.001 for non-inferiority
Sotagliflozin	SOLOIST-WHF (27)	1,222 T2DM patients were hospitalized due to the presence of signs and symptoms of HF and were treated with intravenous diuretic therapy and eGFR≥30 mL/min/1.73 m^2^	9.0 months	The first occurrence of CVD-related death causes or hospitalization for HF 0.67 (0.52–0.85), *P* < 0.001
	SCORED (28)	10,584 T2DM patients with eGFR of 25 to 60 mL/min/1.73 m^2^ and risk factors for cardiovascular disease	16 months	MACE^i^,0.74 (0.63–0.88), *P* < 0.001

a *Death from CVD causes, non-fatal MI, or non-fatal stroke;*

b *eGFR of 30 to <90 mL/min/1.73 m^2^ of body-surface area and albuminuria (UACR, >300 to 5,000, with albumin measured in milligrams and creatinine in grams);*

c *Dialysis, transplantation, or a sustained eGFR of < 15 mL/min/1.73 m^2^;*

d *Death from CVD causes, non-fatal MI, or non-fatal stroke;*

e *CVD death, MI, or ischemic stroke;*

f *Hospitalization or an urgent visit resulting in intravenous therapy for HF;*

g *Maintenance dialysis for ≥28 days, kidney transplantation, or an eGFR of <15 mL/min/1.73 m^2^ confirmed by a second measurement after ≥28 days;*

h *Death from CVD causes, non-fatal MI, or non-fatal stroke;*

i *Death from CVD causes, non-fatal myocardial infarction, or non-fatal stroke*.

*ASCVD, Atherosclerotic cardiovascular disease; CI, Confidence interval; CKD, Chronic kidney disease; CVD, Cardiovascular disease; EF, Ejection fraction; eGFR, Estimated glomerular filtration rate; ESKD End stage kidney disease; HFrEF, Heart failure with reduced ejection fraction; HR, Hazard ratio; MACE, Major adverse cardiac events; MI, Myocardial infarction; NT-proBNP, N-terminal pro-brain natriuretic peptide; T2DM, Type 2 Diabetes Mellitus; UACR, Urinary albumin-to-creatinine ratio*.

Network pharmacology is a discipline based on systems biology and multidirectional pharmacology that uses specific nodes and adopts biomolecular network analysis methods to conduct drug molecular design and target analysis (29). When compared to traditional experimental pharmacology approaches, network pharmacology is based on a complete system and can more effectively investigate the target and pathway relationships between medications and disorders more effectively. Therefore, we utilized the network pharmacology analysis system to determine the targets of SGLT2 inhibitors on the cardiorenal protection of T2DM and identify the biological pathways involved to provide directions for in-depth investigation of SGLT2 inhibitors.

## Methods and Materials

### SGLT2 Inhibitor-Related Targets

The structural formulae of Ertugliflozin, Sotagliflozin, Canagliflozin, Dapagliflozin, Empagliflozin, Tofogliflozin, Luseogliflozin, and Ipragliflozin were obtained from PubChem (https://pubchem.ncbi.nlm.nih.gov/) (30). The structural formulae were added to the SwissTargetPrediction database (https://www.drugbank.com/) with probability>0 screening (31). To predict the targets, the DrugBank (https://www.drugbank.com/) was also utilized to forecast the targets (32). The intersection of the results was used.

### Disease Targets

In order to obtain potential targets, the keywords “Chronic Kidney Disease,” “Heart Failure” and “Type 2 Diabetes Mellitus” were retrieved from Genecards (http://www.genecards.org/), Disgenet (https://www.disgenet.org/), Online Mendelian Inheritance in Man (OMIM, https://omim.org/), Pharmacogenomics Knowledgebase (PharmGKB, https://www.pharmgkb.org/) and Therapeutic Target Database (TTD, http://db.idrblab.net/ttd/).

### Venn Diagram and “Drugs-Targets” Regulatory Network

Venn diagrams were created using R packages (“VennDiagram”) from the intersection of medication targets acquired in item 2.1 and illness targets obtained in item 2.2. The regulatory network was constructed using the Cytoscape3.8.2 software, with SGLT2 inhibitors and intersection targets serving as nodes.

### Protein Protein Interaction (PPI) Network

The common interaction targets were imported into the Search Tool for the Retrieval of Interacting Genes database (STRING, Version 11.5, https://www.string-db.org/), screened with an interaction score ≥0.4, unlinked nodes in the network were hidden, the remaining parameters were left unchanged, and the obtained results were exported in text form into the Cytoscape 3.8.2 software for visualization (33). The Cytohubba and Molecular Complex Detection (MCODE) plug-ins in Cytoscape 3.8.2 were used to filter the modules of the PPI network with high significance. The cytohubba plug-in filtering criterion was to compute the node scores and rank the top 10 significant genes based on the maximal clique centrality (MCC) (34); the MCODE plug-in filtering criterion was degree cutoff = 2, node score cutoff = 0.2, K-score = 2, Max depth = 100, with *P* < 0.05 indicating a statistically significant difference (35). The previously mentioned two intersection targets were chosen after screening the targets separately.

### Analysis of Functional Enrichment Analysis

Gene ontology (GO) categorizes genes into three groups: biological process (BP), cellular component (CC), and molecular function (MF). The Kyoto encyclopedia of genes and genomes (KEGG) was used to investigate the signaling pathways involved in genes. GO and KEGG enrichment analysis visualization is completed using R packages “org.hs.eg. db,” “DOSE,” “clusterProfiler,” “enrichPlot,” “Colorspace,” “stringi” and “GGploT2,” and output the enriched bubble plots in accordance the number and significance of enrichment (*p*-value cutoff <0.05, q-value cutoff <0.05). The obtained KEGG pathway was brought into Cytoscape 3.8.2, and the “pathway-gene” network was built based on the degree value.

### Molecular Docking

Cytohubba and MCODE screened intersecting targets, and the first three core targets in the “pathway-gene” network were molecularly docked to SGLT2 inhibitors. The protein 3D format of the core targets was downloaded from the Protein Data Bank (PDB, https://www.rcsb.org/) (36), and operations such as dehydration, hydrogenation, and ligand extraction were carried out using Pymol 2.5 software (37). The Autodock Vina 1.1.2 program was used for molecular docking (38).

## Results

### Potential SGLT2 Inhibitor-Related Targets

SwissTargetPrediction was utilized to obtain 71 Ertugliflozin targets, 103 Sotagliflozin targets, 53 Canagliflozin targets, 60 Dapagliflozin targets, 17 Empagliflozin targets, 57 Tofogliflozin targets, and 59 Ipragliflozin targets. Eight Canagliflozin targets, 11 Dapagliflozin targets, and 10 Empagliflozin targets were found in Drugbank. After de-duplicating and combining, 164 SGLT2 inhibitor targets were found.

### Drugs-Targets Intersection Targets and Regulatory Network

**Figure 1:**
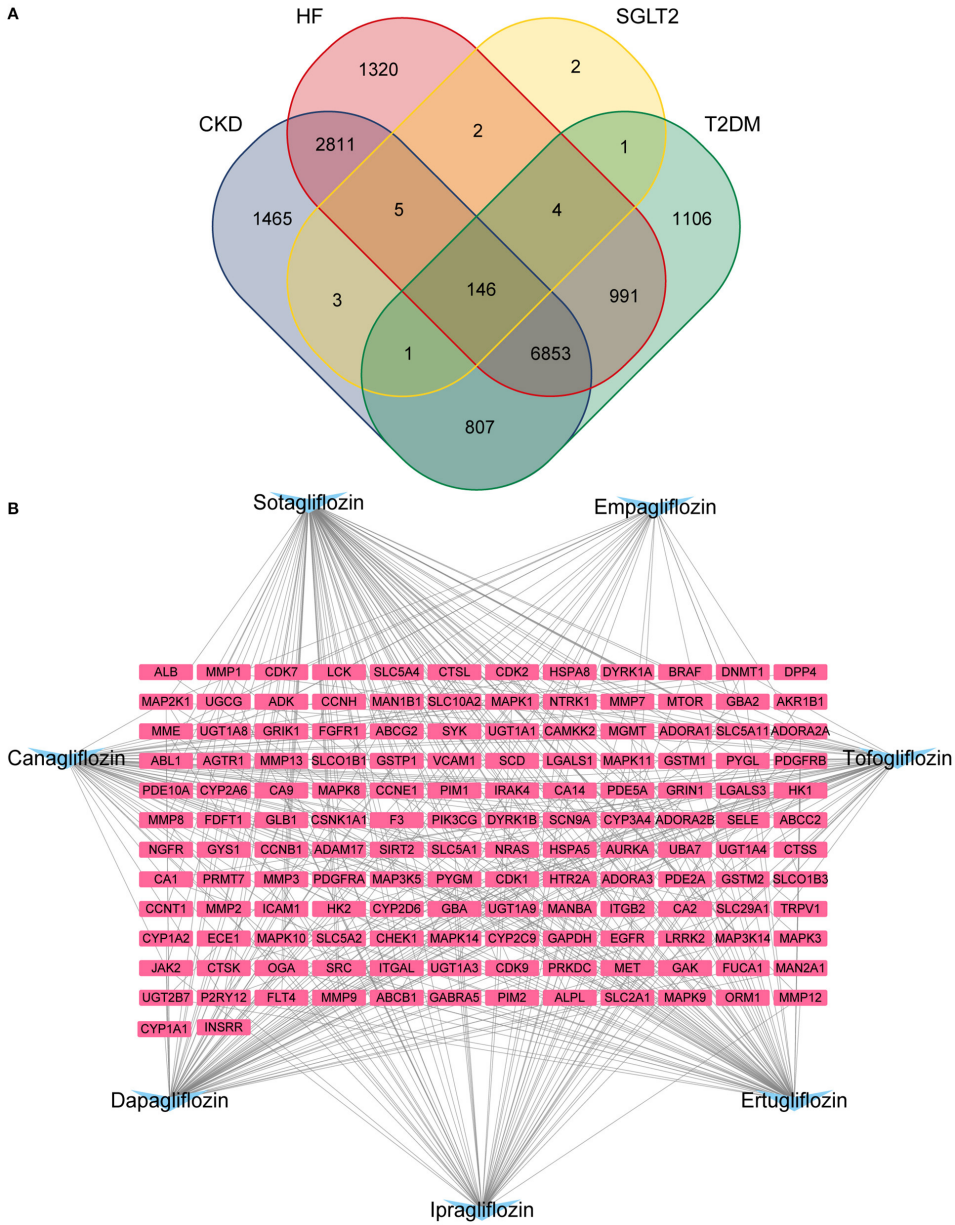
Potential SGLT2 inhibitors-related targets. **(A)** Venn diagram showing that 146 targets were common to CKD, HF, SGLT2, and T2DM; **(B)** Interaction network to indicate drugs-targets composited of SGLT2 inhibitors (blue) and 146 targets (pink).

After de-duplication and merging, a total of 12,132 HF-related targets, 12,091 CKD-related targets, and 9,910 T2DM-related targets were obtained by searching in Genecards, DisGeNet, OMIM, PharmGKB, and TTD databases. A Venn diagram was used to identify the 146 targets of SGLT2 inhibitors that intersected with disease (Figure 1A). The 146 targets were loaded into Cytoscape 3.8.2 to generate a drugs-targets regulatory network with 153 nodes and 409 edges, indicating the interaction between the SGLT2 inhibitors and the putative targets (Figure 1B).

### Building a PPI Network and Screening Key Targets

**Figure 2:**
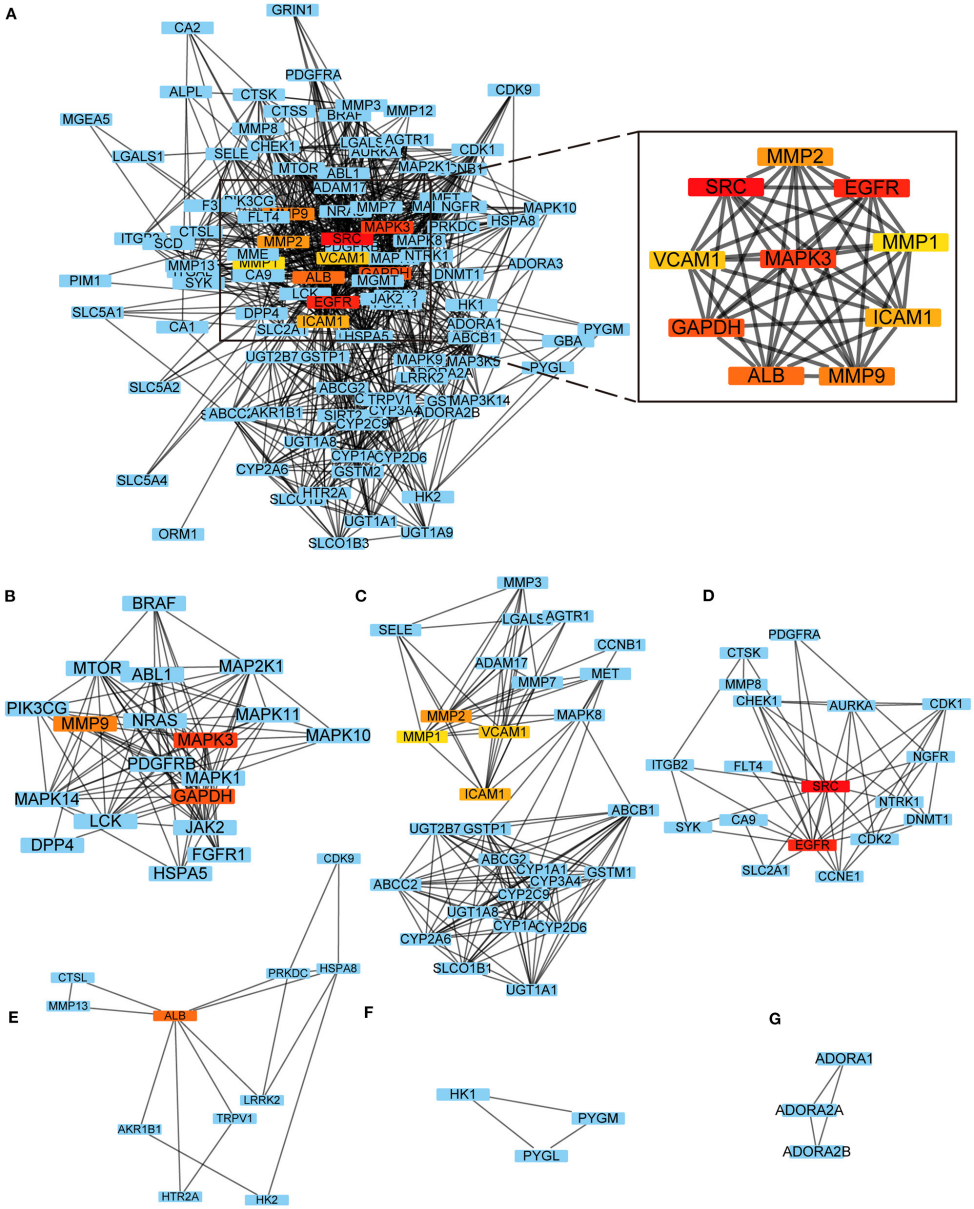
PPI network. **(A)** Top 10 key targets in the PPI network obtained by the Cytohubba; **(B–G)** The results of the MCODE plug-in clustering analysis.

The PPI network had 1,049 pairs of interactions, and the top 10 key targets in the PPI network obtained by the Cytohubba plug-in were: SRC, EGFR, MAPK3, GAPDH, ALB, MMP9, MMP2, ICAM1, VCAM1, and MMP1 (Figure 2A); the detailed results of the MCODE plug-in clustering analysis are depicted in Table 2, and Figures 2B–G; the key targets of their intersection are GAPDH, MAPK3, and MMP9.

**Table 2:** Cluster details based on MCODE plug-in.

**Clusters**	**Targets contained in clusters**	**Score**	**Nodes**	**Edges**
1	MAPK14, PIK3CG, MAPK1, HSPA5, DPP4, BRAF, FGFR1, MMP9, JAK2, ABL1, MAPK3, MTOR, MAPK10, NRAS, MAP2K1, LCK, MAPK11, GAPDH, PDGFRB	11.111	19	100
2	CYP1A1, UGT1A1, MMP1, ABCC2, MMP3, CYP2D6, VCAM1, MET, CYP1A2, ICAM1, CYP3A4, AGTR1, GSTM1, MMP7, UGT1A8, UGT2B7, MMP2, SELE, ADAM17, CYP2A6, CCNB1, CYP2C9, LGALS3, SLCO1B1, ABCG2, ABCB1, GSTP1, MAPK8	10.667	28	144
3	FLT4, NGFR, SRC, NTRK1, DNMT1, EGFR, PDGFRA, CDK1, AURKA, CA9, CHEK1, CTSK, ITGB2, SLC2A1, CDK2, SYK, CCNE1, MMP8	5.294	18	45
4	HK2, HTR2A, TRPV1, ALB, HSPA8, MMP13, AKR1B1, PRKDC, CDK9, LRRK2, CTSL	3.4	11	17
5	PYGM, HK1, PYGL	3	3	3
6	ADORA2B, ADORA2A, ADORA1	3	3	3

### Analysis of GO and KEGG Enrichment

**Figure 3:**
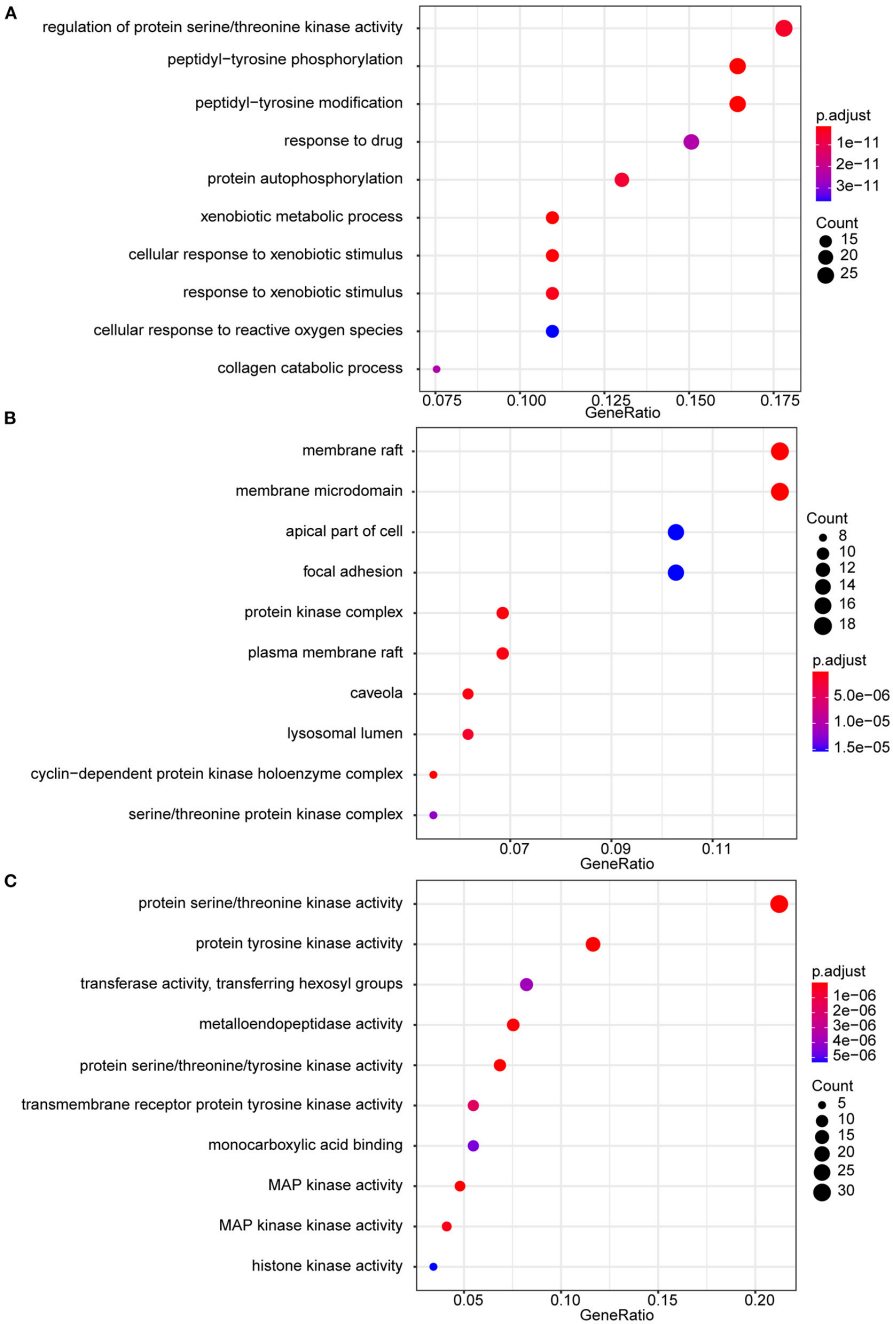
GO enrichment analysis. **(A)** GO biological processes of 146 core genes; **(B)** GO cellular component of 146 core genes; **(C)** GO molecular function f 146 core genes.

GO functional analysis was performed on 146 intersecting targets, and the results were screened at *P* < 0.05. The enrichment results revealed a total of 1,089 BP, which mainly involved regulating protein serine/threonine kinase activity, peptidyl-tyrosine phosphorylation, and peptidyl-tyrosine modification. The overall number of CC was 66, with most of them relating to membrane raft and membrane microdomains. The total number of MF included 145, with protein serine/threonine kinase activity and protein tyrosine kinase activity. Figure 3 shows the top 10 items in the BP, CC, and MF categories.

**Figure 4:**
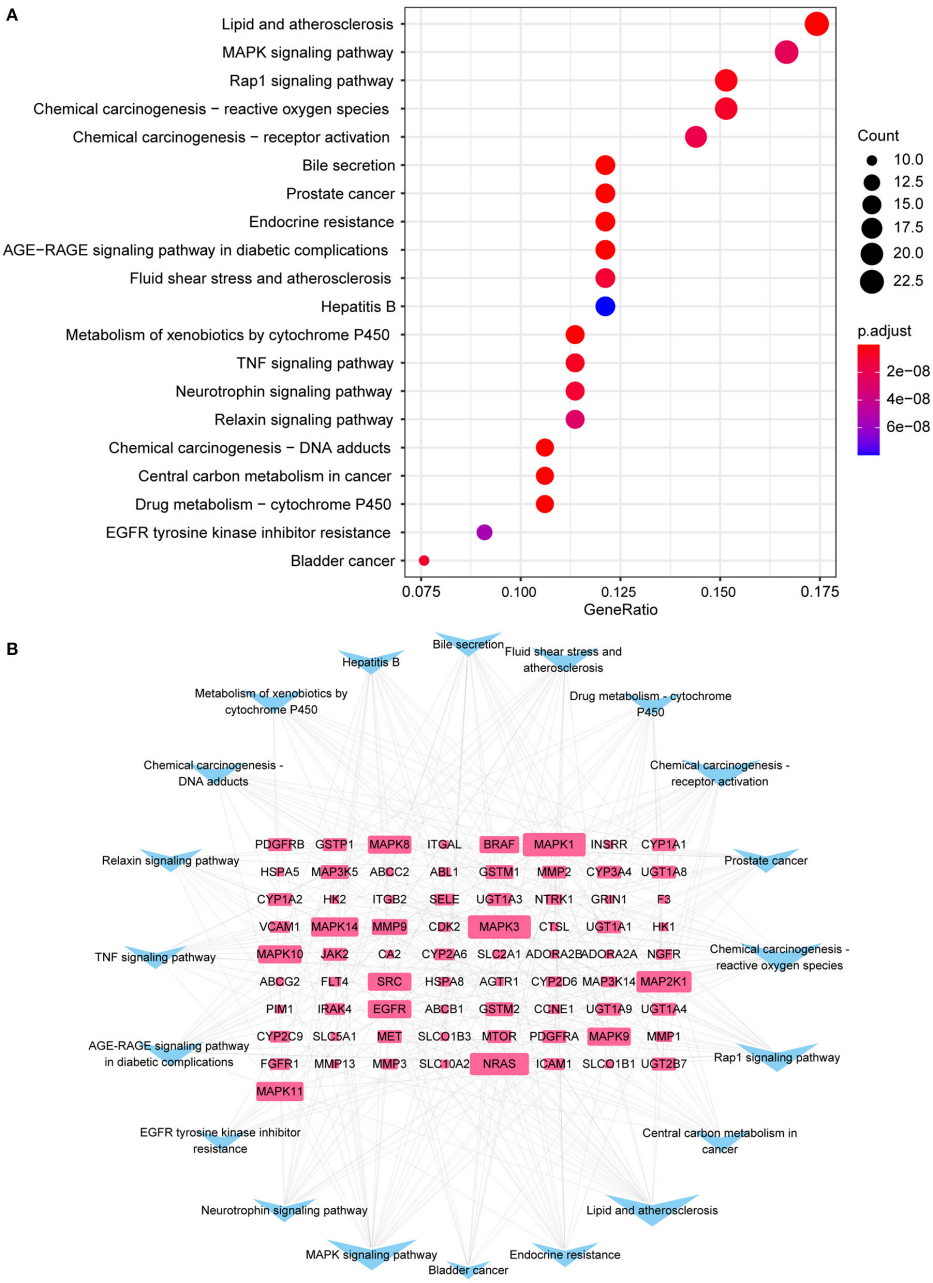
KEGG enrichment analysis. **(A)** The top 20 enriched pathways; **(B)** Interaction network of 73 targets (blue) and top 20 pathways (pink) to indicate pathways-targets network.

The cardiorenal protective effect of SGLT2 inhibitors may be related to 147 KEGG signaling pathways in diabetic complications, including lipid and atherosclerosis, MAPK signaling pathway, Rap1 signaling pathway, endocrine resistance, fluid shear stress, and atherosclerosis, TNF signaling pathway, relaxin signaling pathway, neurotrophin signaling pathway, and AGEs-RAGE signaling pathway (Figure 4A). The top 20 enriched pathways were imported into Cytoscape 3.8.2 to build a pathways-targets network, and the results revealed that the top 10 targets associated with the enriched pathway are MAPK1, MAPK3, NRAS, MAP2K1, MAPK11, MAPK14, MAPK10, MAPK8, and MAPK9 (Figure 4B).

### Molecular Docking

**Figure 5:**
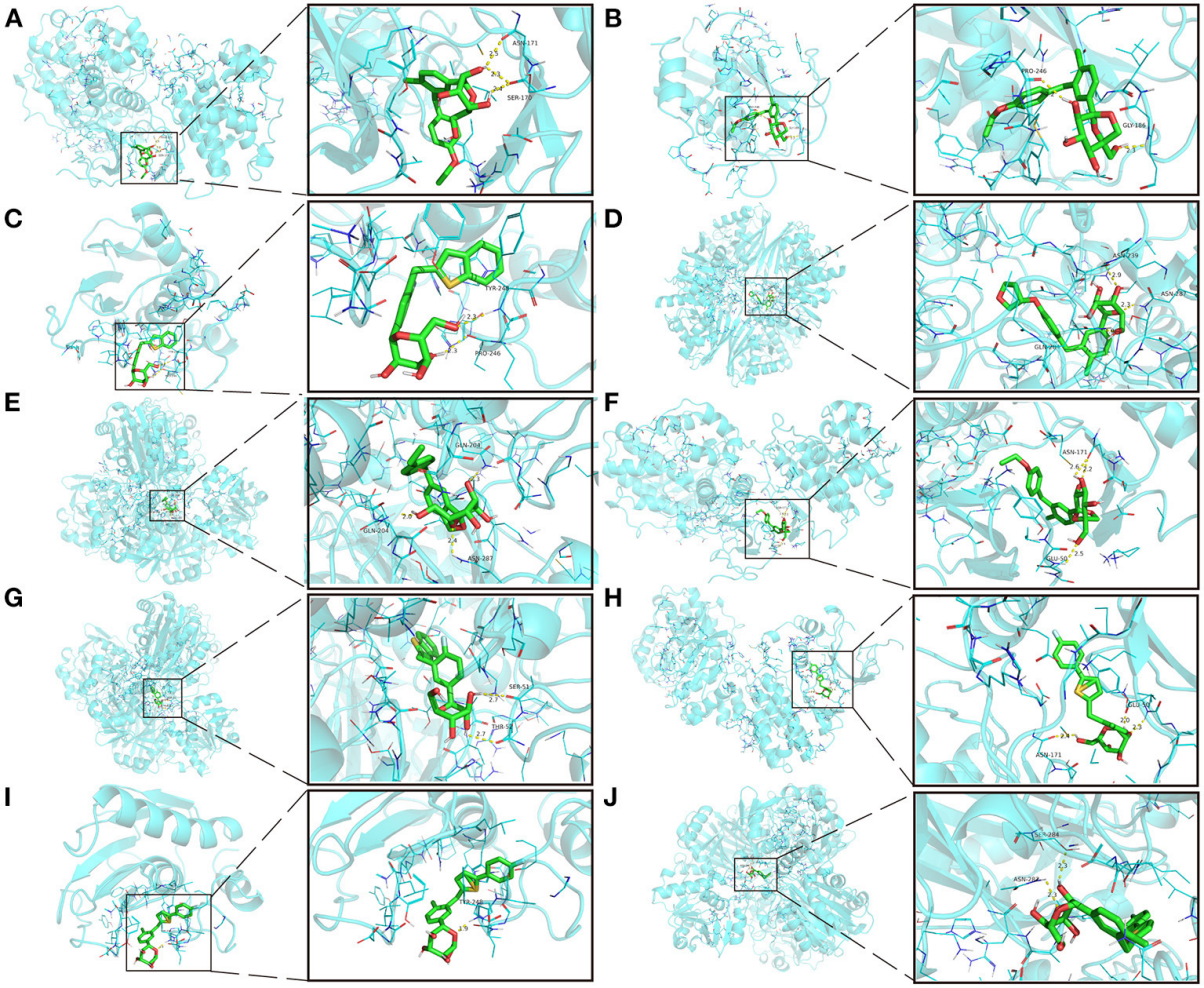
The top 10 docking results. **(A)** Dapagliflozin-MAPK3; **(B)** Ertugliflozin-MMP9; **(C)** Ipragliflozin-MMP9; **(D)** Empagliflozin-GAPDH; **(E)** Tofogliflozin-GAPDH; **(F)** Ertugliflozin-MAPK3; **(G)** Ipragliflozin-GAPDH; **(H)** Canagliflozin-MAPK3; **(I)** Canagliflozin-MMP9; **(J)** Canagliflozin-GAPDH. The dashed lines in the graph are hydrogen bonds and the values are bond lengths. The names represent residues in the binding sites.

The intersection targets of PPI network and pathways-targets network GAPDH, MAPK3, MMP9, MAPK1, and NRAS were docked with SGLT2 inhibitors. Binding energy <0 indicates that the ligand and receptor are spontaneous bindings, and the lower the affinity value in the docking result, the more stable the interaction between the target and the active ingredient. Molecular docking revealed that the five important targets mentioned above have good binding activity to SGLT2 inhibitors (Table 3). The top 10 docking results were selected for visual analysis (Figure 5). The dashed lines in the graph are hydrogen bonds and the values are bond lengths. The names represent residues in the binding sites.

**Table 3:** Molecular docking results of SGLT2 inhibitors.

**SGLT2 inhibitors**	**Structure**	**PubChem CID**	**Target**	**PDB ID**	**Binding energy (kcal/mol)**
Canagliflozin	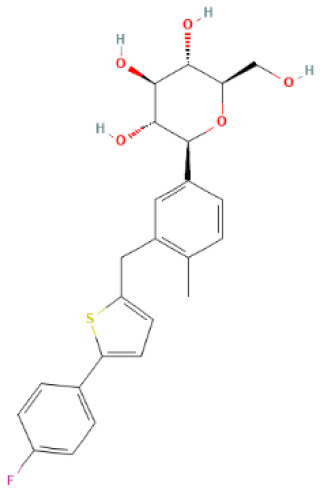	24812758	GAPDH	1U8F	−11
			MAPK3	4QTB	−10
			NRAS	5UHV	−8.9
			MMP9	6ESM	−10.3
			MAPK1	6SLG	−8.9
Dapagliflozin	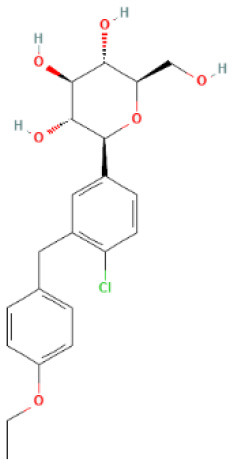	9887712	GAPDH	1U8F	−8.9
			MAPK3	4QTB	−9.3
			NRAS	5UHV	−7.5
			MMP9	6ESM	−7.9
			MAPK1	6SLG	−8
Empagliflozin	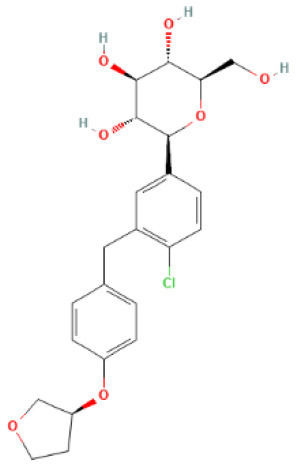	11949646	GAPDH	1U8F	−9.7
			MAPK3	4QTB	−8.8
			NRAS	5UHV	−8
			MMP9	6ESM	−9.1
			MAPK1	6SLG	−8.4
Ertugliflozin	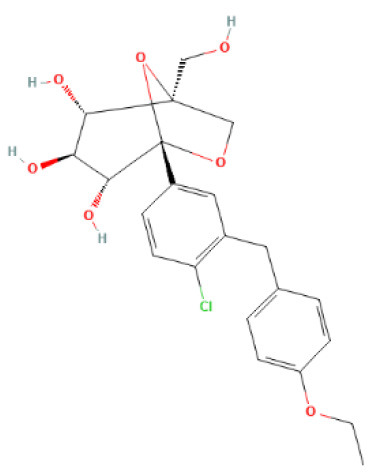	44814423	GAPDH	1U8F	−9.3
			MAPK3	4QTB	−9.8
			NRAS	5UHV	−7.8
			MMP9	6ESM	−9.4
			MAPK1	6SLG	−8.3
Ipragliflozin	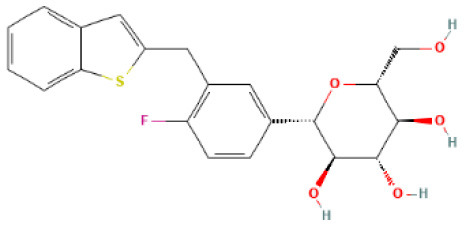	10453870	GAPDH	1U8F	−9.9
			MAPK3	4QTB	−9.3
			NRAS	5UHV	−8.6
			MMP9	6ESM	−9.6
			MAPK1	6SLG	−8.4
Sotagliflozin	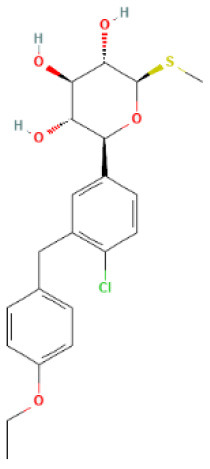	24831714	GAPDH	1U8F	−7.3
			MAPK3	4QTB	−7.7
			NRAS	5UHV	−7.2
			MMP9	6ESM	−8
			MAPK1	6SLG	−7.8
Tofogliflozin	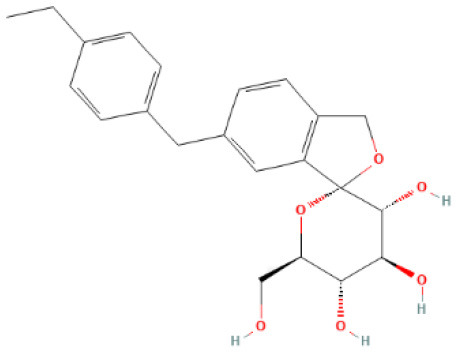	46908929	GAPDH	1U8F	−9.7
			MAPK3	4QTB	−8.2
			NRAS	5UHV	−8
			MMP9	6ESM	−8.5
			MAPK1	6SLG	−8.4

## Discussion

The main complications of T2DM are HF and CKD, and the three can interact. Patients with T2DM develop a variety of cardiac abnormalities, a phenomenon known as “diabetic cardiomyopathy” (39). Furthermore, the accumulation of AGEs due to hyperglycaemia can lead to microvascular remodeling and cardiac fibrosis (40). The primary mechanism for the progression of HF stems from the stimulation of the renin-angiotensin-aldosterone system (RAAS) (41), and excessive activation of the RAAS can interfere with insulin production, secretion, and metabolic pathways, leading to reduced insulin sensitivity and disturbed glucose metabolism (42). These metabolic changes may not only impair myocardial energetics and reduce the efficiency of mechanical work but also promote systemic insulin resistance, creating a vicious cycle in which HF causes metabolic modifications and thus inducing HF (43). The multiple metabolic and haemodynamic alterations caused by T2DM promote structural changes in the kidney, primarily by affecting microcirculation. These changes further activate harmful pathways (e.g., inflammation), leading to glomerular fibrosis and tubular atrophy and eventually to end-stage renal disease (44). Clinically and pathophysiologically, HF and CKD are significantly correlated, and this association is especially pronounced in patients with coexisting T2DM (45). Haemodynamic changes owing to decreased cardiac output and/or altered venous return, sympathetic activation, and/or stimulation of the RAAS controlling the (neuro)hormonal axis are all pathophysiological processes that contribute to the genesis and progression of cardio-renal and renal-heart interactions. In addition to other factors that accelerate the progression of HF and CKD (local and systemic inflammation, metabolic changes, anemia, etc.) (46). With the progression of clinical studies with SGLT2 inhibitors, the results suggest a cardiorenal protective effect in patients with or without DM. Although several hypotheses have been proposed, the mechanisms are unknown; hence, we used a network pharmacology method to investigate the mechanisms of cardiorenal protection.

In the regulatory network, there are 7 SGLT2 inhibitors, namely Ertugliflozin, Sotagliflozin, Canagliflozin, Dapagliflozin, Empagliflozin, Tofogliflozin, and Ipragliflozin. Sotagliflozin is an SGLT2 inhibitor with minor SGLT1 inhibitory activity (47). Several recent studies have revealed potential mechanisms of SGLT2 inhibitors. DM induces reactive oxygen species (ROS), apoptosis, and endoplasmic reticulum (ER) stress, which could be reversed with Dapagliflozin therapy (48). The renoprotective effects of dapagliflozin in prediabetes by alleviating obesity-induced renal inflammation, fibrosis, ER stress, apoptosis, and lipid accumulation (49). Dapagliflozin also slowed the progression of diabetic kidney disease by decreasing cellular senescence and oxidative stress via ketone-induced nuclear erythroid-related factor 2 (NRF2) activation (50). The vasoprotective effect of dapagliflozin may be due to improved transport of bone marrow-derived haematopoietic cells to the site of vascular injury (51). Empagliflozin-associated renoprotection in injured proximal tubules of mice with apolipoprotein E (APOE) deficiency given a high-fat diet is mediated by increased ketone body, which corrects mechanistic target of rapamycin complex 1 (mTORC1) hyperactivation detected in non-proteinuric and proteinuric diabetic kidney disease (52). The etiology of HF is linked to the activation of the late component of the cardiac sodium channel current (late-INa), which is a molecular target for Empagliflozin (53). Empagliflozin's prevention of HF progression is linked to reduced proximal tubule sodium-hydrogen exchanger 3 (NHE3) activity, euvolemia restoration, and renal mass maintenance (54). A study on human cardiac microvascular endothelial cells in a co-culture system with adult rat ventricular cardiomyocytes to measure diastolic and systolic function revealed that serum from CKD patients impaired the beneficial effects of cardiac microvascular endothelial cells on cardiomyocyte function and that Empagliflozin restored this effect by reducing mitochondrial oxidative damage (55). Reversion-inducing-cysteine-rich protein with Kazal motifs (RECK), a membrane-anchored matrix metalloproteinase (MMP) regulator, is linked to fibrosis. Empagliflozin reduces systemic and renal arterial stiffness and reverses RECK expression in T2DM female mice, alleviating kidney damage (56).

The key targets obtained by the PPI network are 3-phosphoglyceraldehyde dehydrogenase (GAPDH), mitogen-activated protein kinase 3 (MAPK3), MAPK1, NRAS and MMP9 combined with KEGG enrichment results. GAPDH is a simple “housekeeping” protein that plays a role in a variety of physiological and pathological processes and has a variety of non-glycolytic functions in addition to its part in glycolysis (57). These multifunctional properties of GAPDH may depend on its posttranslational modification, oligomerization and significant subcellular localizations (including cytoplasm, nucleus, mitochondria and vesicles) (58). The significant expression of GAPDH in the cytoplasm may enable it to operate as an intracellular sensor capable of directly transmitting signals to numerous organelles, such as the mitochondria. GAPDH translocation to mitochondria has been proven to cause the inner transmembrane potential to be lost, the matrix to swell, and the inner mitochondrial membrane to permeabilize (59). These changes lead to apoptosis, which is closely associated with CKD and HF (60, 61). Hyperglycemia, on the other hand, causes an excess of acetoacetyl coenzyme A, which feeds into the tricarboxylic acid cycle, resulting in an excess of nicotinamide adenine dinucleotide (NADH), putting the mitochondrial electron transport chain under a lot of stress (62). Thus, mitochondrial oxidation of overproduced NADH will eventually result in the creation of more superoxide and hence more ROS, which can target and inactivate GAPDH (63). This would cause a buildup of glycolytic metabolites upstream of glyceraldehyde 3-phosphate as well as the activation of alternate glucose disposal pathways, all of which are associated with ROS generation and hence enhance oxidative stress (9). Dapagliflozin has been proven in studies to alleviate glucotoxicity by lowering excessive glucose influx into renal tubular epithelial cells under high glucose circumstances through activating GAPDH (64). Furthermore, dapagliflozin alleviates pressure overload-induced myocardial remodeling in mice via inhibiting ER stress-mediated apoptosis (65). Empagliflozin improved renal ischemia/reperfusion injury in non-diabetic rats by promoting mitochondrial biogenesis and inhibiting oxidative stress and apoptosis (66). In the murine model of left ventricular pressure overload, empagliflozin aids the suppression of endothelial apoptosis, the maintenance of capillarization, and the improvement of cardiac systolic dysfunction (67). MAPK3/MAPK1, also known as extracellular signal-regulated kinase 1/2 (ERK1/2), is a member of the MAPK protein family (68). MAPKs are serine/threonine protein kinases that phosphorylate a varied array of downstream targets in distinct compartments in order to carry out highly specific physiological responses in response to activation (69). MAPK signaling has four major branching routes, including ERK1/2, which is often mitogen sensitive but also the most widely responsive to a large range of stimuli; ERK5, which is functionally similar to ERK1/2; and the c-Jun N-terminal kinases (JNK) as well as p38 families, which are activated by environmental stress changes and inflammatory factors (70). ERK1/2 is the most critical effector in cardiomyocytes, particularly cardiac hypertrophy, and has also been identified as a key component of the cardioprotective reperfusion damage salvage kinase pathway (71, 72). In adult organs, ERK1/2 mediates transcription and post-transcriptional regulation of numerous nephron channels and transporters, resulting in acid-base and electrolyte balance stabilization (73). ERK1/2 s also closely related to the pathogenesis of hypoxia-induced renal fibrosis (74). Pretreatment with empagliflozin protects the heart from I/R injury-induced severe fatal ventricular arrhythmia, which might be the result of glucose-independent stimulation of the ERK1/2 (75). Canagliflozin, not empagliflozin or dapagliflozin, alleviates inflammation and lowers Hexokinase II expression by inhibiting ERK1/2 phosphorylation in lipopolysaccharide-stimulated human coronary artery endothelial cells (76). Neuroblastoma RAS Viral Gene Homolog (NRAS) is a RAS family member that regulates the MAPK signaling pathway. Because it has been examined extensively in tumor illnesses, it is rarely discussed (77, 78). Both CKD and HF are associated with changes in the extracellular matrix (ECM), and MMPs are primarily responsible for ECM degradation (79, 80). MMP9, also known as gelatinase B, can degrade elastin and collagen and is involved in inflammatory responses, and is one of the most extensively studied MMPs in cardiovascular and renal research (81, 82). Low-dose empagliflozin can improve systolic heart function after MI in rats by regulating MMP9 (83). Luseogliflozin regulates the expression of multiple mRNA, including MMP9, and inhibits the progression of atherosclerosis in diabetic APOE-deficient mice (84).

The results of GO functional enrichment are primarily related to protein serine/threonine kinase activity. Protein kinases and protein phosphatases maintain the balance of protein phosphorylation regulation as two major classes of proteins, phosphorylation, and dephosphorylation, respectively. Phosphorylation of proteins occurs most commonly at serines and threonines, where protein kinases transfer phosphate groups to these amino acid residues, usually using adenosine triphosphate as a substrate, and protein phosphatases hydrolyze phosphate from the amino acid residues of the protein. They collaborate to regulate protein function, stability, and signaling, as well as carrying out physiopathological tasks (85). Protein kinase C (PKC) isozymes are a group of serine/threonine kinases whose activity and levels are dispensed in several pathological heart conditions, including atherosclerosis (AS), myocardial infarction (MI), acute ischaemic, cardiac hypertrophy, cardiac arrhythmia, HF, and cardiac fibrosis (86, 87). The AKT (also known as protein kinase B, PKB) family also belongs to serine/threonine protein kinases, and its activation is critical in a variety of signaling pathways linked to kidney injury (88). Some serine/threonine kinases, such as Rho-associated, coiled-coil containing kinases (ROCK), have been implicated in cardiovascular–renal remodeling via oxidative stress and oxidative stress-related signaling via nicotinamide adenine dinucleotide phosphate (NADPH) oxidase induction (89).

According to the KEGG enrichment results, SGLT2 inhibitors' cardiorenal protection may be associated with Lipid and AS, MAPK signaling pathway, Ras-related Protein 1 (Rap1) signaling pathway, Endocrine resistance, Fluid shear stress, and atherosclerosis, tumor necrosis factor (TNF) signaling pathway, Relaxin signaling pathway, Neurotrophin signaling pathway, Advanced glycation end-products (AGEs)- receptor for advanced glycation end-products (RAGE) signaling pathway in diabetic complications. AS is a slow-progressing inflammatory illness that is a primary underlying pathology for CVD, including MI, HF, stroke, and peripheral artery disease, which are the top causes of mortality in DM and CKD patients (90). DM is a risk factor for AS, and CKD can also accelerate AS through increased inflammation, lipid metabolism disorders, and other mechanisms (91). According to the findings of a systematic review and meta-analysis, glucose-lowering medications, such as SGLT2 inhibitors, can greatly reduce the risk events of AS (92). A wide range of multinational observational studies in individuals with T2DM and cardiovascular risk has also demonstrated that the beneficial effects of SGLT2 inhibitors are associated with MI and stroke, with AS and its clinical consequences being the most closely linked (93, 94). The results of network pharmacology suggest a mechanism of SGLT2 associated with AS, which is supported by experimental evidence. Canagliflozin, for example, in the APOE knockout mice model, could slow the progression of AS by decreasing the expression of inflammatory molecules such as monocyte chemoattractant protein-1 (MCP-1) and vascular cell adhesion molecule-1 (VCAM-1). Additionally, it could increase the stability of atherosclerotic plaques by increasing the tissue inhibitor of metalloproteinase 1 (TIMP1)/MMP2 ratio (95). Empagliflozin has been shown to reduce lipid profiles and sympathetic activity in AS (96). ERK1/2 in the MAPK signaling pathway have been mentioned above, and ERK5 has a similar function with relatively fewer studies, but it also plays an important role. ERK5 is a protein that is predominantly located in the glomerular mesangium and is expressed in the kidney. It is involved in the contraction, proliferation, and ECM accumulation of disease-induced mesangial cells (97, 98). The use of lentivirus to overexpress full-length ERK5 in the kidneys of mice protected them from renal I/R injury (99). Targeted ERK5 deletion reduces hypertrophy and increases pressure overload-induced apoptosis in the heart (100). In female mice, ERK5 regulates body weight and systemic energy balance, most likely through its expression in hypothalamus neurons, making it a target for metabolic illnesses such as T2DM (101). The involvement that JNK plays in cardiac hypertrophy and I/R is less clear because JNK activation is most likely a dynamic signal transduction event that can be modified by the nature of the stimuli and that different JNK (JNK1-3) isoforms may play separate roles in the process (102–104). In addition, reduced myocardial connexin 43 expression and gap junction integrity, caused by the activation of the JNK signaling pathway, have been associated with conduction abnormalities and abrupt HF in mice (105). The JNK pathway can be triggered by a variety of stimuli associated with acute and chronic kidney injury, including pro-inflammatory cytokines, pro-fibrotic factors, risk-associated molecular pattern ligands, oxidative stress and nephrotoxins. The activated JNK pathway may either promote renal fibrosis through its pro-apoptotic and pro-inflammatory effects or directly enhance the fibrotic response (106). JNK also interacts with other pro-fibrotic pathways, including the transforming growth factor-β (TGF-β)/small mothers against decapentaplegic SMAD pathway (107). JNK is also involved in the regulation of insulin resistance and β-cell dysfunction (108). The quantity, length, mode, and timing of induction involving different isoforms (α, β, γ, and δ) and upstream/downstream pathways determine the specific role of p38 in myocardial ischemia damage and protection (109). Acute p38 signaling activation may serve as an adaptive response to extracellular stresses in the early phases of hypertrophy, but prolonged p38 signaling activation appears to have deleterious implications, including suboptimal cardiac remodeling and HF (110). The majority of evidence suggests that p38 activation has a deleterious influence on the onset of HF. The mechanisms at work include the formation of cardiac fibrosis, alterations in Ca^2+^ handling proteins, and gap junction regulation in cardiomyocytes (111). Activation of P38 in intrinsic renal cells (endothelial cells, podocytes and tubular cells) and infiltrating leukocytes is associated with renal dysfunction and histopathology, and plays an important pathogenic role in human glomerulonephritis, contributing to the development of CKD (112). Another interesting finding was the occurrence of renal dysfunction and fibrosis and the activation of P38 in both cerebral infarction and MI mouse models, suggesting that P38 plays a key role in the fibrotic response induced by signals from different injury modes (113, 114). In RAW 264.7 macrophages stimulated by lipopolysaccharide, empagliflozin had no effect on phosphorylation of p38 and ERK, but it did reduce phosphorylation of JNK (115). Empagliflozin reduces high glucose-induced RECK suppression, oxidative stress and epithelial-to-mesenchymal transition in cultured kidney proximal tubule cells and these beneficial effects are partially associated with p38 (116). Empagliflozin also attenuates the aging of cardiac stromal cells and improves heart function in a diabetic mouse model by targeting P38 (117). Canagliflozin protects endothelium function in APOE -deficient mice via inhibiting P38 activation (118). MAPK signaling plays a critical and complicated role in cardiorenal protection, which can only be briefly summarized here. The protective mechanism of SGLT2 seems to be more related to the ERK1/2 and P38 pathways. Rap1 is a small molecular weight GTPase that belongs to the Ras family. On the one hand, it maintains telomere function in the nucleus, and on the other hand, the Rap1 signaling pathway is primarily involved in cell adhesion, cell connection formation, and endothelial barrier protection (119). Rap1 is required to form a podocyte slit diaphragm, a specialized junction universally injured in proteinuric diseases and as an intracellular signaling hub (120). In rats with streptozotocin-induced diabetes, Rap1 ameliorates tubular damage in diabetic nephropathy by modulating primarily the mitochondrial-derived oxidative stress (121). In the cardiovascular system, Rap1 signaling promotes angiogenesis and maintenance of vascular stability by promoting vascular endothelial growth factor receptor 2 (VEGFR2) activation or acting downstream from vascular endothelial growth factor (VEGF), fibroblast growth factor 2 (FGF2), and sphingosine 1-phosphate (S1P) receptors in signaling pathways. Rap1 also regulates cell-cell junctions and heart contractility, but there is little evidence that Rap1 directly promotes hypertrophy (122). Rap1 has been linked to pathologies like metabolism, inflammation and oxidative stress, and regulation of telomeric length, and it may play an important role in diabetic cardiomyopathy (123). Rap1 can also mediate the MAPK pathway through ERK.

Tumor necrosis factor (TNF) is a tiny molecular protein secreted by monocyte-macrophages, including both TNF-α and TNF-β, that binds to two TNF receptors (TNFR) on the cell surface. Besides, it is a cytokine with inflammatory mediator effects (124). Either type of HF is associated with activation of inflammatory signaling (125), and TNF-α-mediated unfavorable remodeling and development of HF may entail impacts on cardiomyocytes, macrophages, and the ECM (126). TNF induces cardiomyocyte apoptosis by activating multiple cell death pathways (127), and RNA sequencing of HF cardiac fibroblasts indicated that TNF-signaling pathways were connected to highly modulated genes involved in ECM structure (128). There is growing evidence that TNF contributes significantly to the pathogenesis of CKD, promoting inflammation, apoptosis, and ECM accumulation, reducing glomerular blood flow, and destroying the glomerular permeability barrier as proteinuria progresses (129). Soluble TNFR-1, the circulating version of the membrane-bound receptor, is correlated with a long-term decline in kidney function for a decade in a multiethnic population (130). SGLT2 inhibitors have been shown to decrease TNF-α, TNF-β or TNFR expression in patients and experimental models (131–133). AGEs are a kind of chemical created by the non-enzymatic interaction between proteins and sugar residues known as glycation or the Maillard reaction (134). AGEs accumulate in the body with age, and DM accelerates this process. The accumulation of AGEs may contribute to the development of HF and CKD by causing protein modifications and triggering numerous inflammatory responses, oxidative stress, and enhancing fibrosis via receptors for advanced glycation end-products (135, 136). Dapagliflozin protects podocytes from advanced AGEs via the adenosine monophosphate-activated protein kinase (AMPK)/mammalian target of rapamycin (mTOR)-mediated autophagy pathway (137). Empagliflozin inhibits the AGEs-receptor axis, which contributes to its anti-inflammatory and antifibrotic actions in experimental diabetic nephropathy (138). Relaxin is a naturally occurring peptide hormone that produces nitric oxide, inhibits endothelin and angiotensin II, produces VEGF and MMPs, and acts as a pleiotropic vasodilator with several pleiotropic effects for HF treatment (139). Several studies have identified the relaxin signaling pathway as a potential therapeutic target for heart failure with preserved ejection fraction (HFpEF) (140, 141). The relaxin signaling pathway also can attenuate tubular epithelial cell apoptosis and prevent renal interstitial fibrosis (142, 143). The neurotrophins family of dimeric polypeptides includes nerve growth factor (NGF), brain-derived neurotrophic factor (BDNF), neurotrophin 3, and neurotrophin 4/5 (144). Even though neurotrophins are well-recognized for their effects on neuronal survival, they also exert cardiovascular effects under physiological and pathological conditions, including pro-angiogenic effects, promoting cardiomyocyte survival, and affecting cardiac sympathetic and parasympathetic nerve activity (145, 146). BDNF effectively repairs podocyte damage by microRNA-134 and microRNA-132 mediated increase in actin polymerization (147). Reduced renal function usually leads to significant disturbances in hormone levels, while HF is strongly associated with insulin resistance, and the potential impact of SGLT2 inhibitors on the heart and kidneys may lie in improving insulin resistance (148, 149).

Network pharmacology is based on a variety of databases and analysis software that can predict the most probable protein targets of SGLT2 inhibitors, thus obtaining the key links in their mechanism of action. However, the main limitation of web-based pharmacology studies is the lack of experimental validation. Pharmacological research will thus be critical in further clarifying the cardiorenal protective effects of SGLT2 inhibitors.

## Conclusion

Clinical research is focused on cardiac and renal protection, regardless of whether DM is comorbid or not. Therefore, we have constructed a network between SGLT2 inhibitors and targets related to CKD, T2DM, and HF and analyzed the potential mechanisms of SGLT2 inhibitors' cardioprotective effects through network pharmacology, which contributes to deepening the understanding of SGLT2 and provide a theoretical for future studies.

## Data Availability Statement

The datasets presented in this study can be found in online repositories. The names of the repository/repositories and accession number(s) can be found in the article/supplementary material.

## Author Contributions

AW: conceptualization, methodology, validation, formal analysis, investigation, data curation, writing—original draft, and visualization. ZL and SZ: conceptualization, methodology, and writing—review and editing. FG: investigation, formal analysis, and data curation. HZ: investigation and data curation. ZZ: formal analysis and visualization. GR: writing–review and editing and supervision. XM: conceptualization, writing–review and editing, supervision, and project administration. All authors contributed to the article and approved the submitted version.

## Funding

This work was supported by the National Key Research and Development Program of China (No. 2018YFC1707410-02). The funder had no role in the study design, data analysis, or decision to publish.

## Conflict of Interest

The authors declare that the research was conducted in the absence of any commercial or financial relationships that could be construed as a potential conflict of interest.

## Publisher's Note

All claims expressed in this article are solely those of the authors and do not necessarily represent those of their affiliated organizations, or those of the publisher, the editors and the reviewers. Any product that may be evaluated in this article, or claim that may be made by its manufacturer, is not guaranteed or endorsed by the publisher.

## Abbreviations

AGEs: Advanced glycosylation end products
AKT/PKB: Protein kinase B
AS: Atherosclerosis
AMPK: Adenosine monophosphate-activated protein kinase
APOE: Apolipoprotein E
BDNF: Brain-derived neurotrophic factor
BP: Biological process
CC: Cellular component
CKD: Chronic kidney disease
CVD: Cardiovascular disease
DM: Diabetes mellitus
ECM: Extracellular matrix
ER: Endoplasmic reticulum
ERK1: Extracellular signal-regulated kinase 1
FGF2: Fibroblast growth factor 2
GAPDH: 3-phosphoglyceraldehyde dehydrogenase
GO: Gene ontology
H9C2: Rat cardiomyocytes
HF: Heart failure
HFpEF: Heart failure with preserved ejection fraction
JNK: c-Jun N-terminal kinases
KEGG: Kyoto encyclopedia of genes and genomes
MAPK: Mitogen-activated protein kinase
MCC: Maximal clique centrality
MCODE: Molecular Complex Detection
MCP-1: Monocyte chemoattractant protein-1
MF: Molecular function
MI: Myocardial infarction
MMP: Matrix metalloproteinase
mTOR: Mammalian target of rapamycin
mTORC1: Mechanistic target of rapamycin complex 1
NADH: Nicotinamide adenine dinucleotide
NADPH: Nicotinamide adenine dinucleotide phosphate
NRAS: Neuroblastoma RAS Viral Gene Homolog
NGF: Nerve growth factor
NHE3: Sodium-hydrogen exchanger 3
NRF2: Nuclear erythroid-related factor 2
OMIM: Online Mendelian Inheritance in Man
PDB: Protein Data Bank
PharmGKB: Pharmacogenomics Knowledgebase
PKC: Protein kinase C
PPI: Protein protein interaction
RAAS: Renin-angiotensin-aldosterone system
RAGE: Receptor for advanced glycation end-products
Rap1: Ras-related Protein 1
RECK: Reversion-inducing-cysteine-rich protein with Kazal motifs
ROCK, Rho-associated: coiled-coil containing kinases
ROS: reactive oxygen species
SAMD: small mothers against decapentaplegic
S1P: Sphingosine 1-phosphate
SGLT2: Sodium-glucose cotransporter 2
STRING: Search Tool for the Retrieval of Interacting Genes database
T2DM: Type 2 Diabetes Mellitus
TGF-β: Transforming growth factor-β
TIMP-1: Tissue inhibitor of metalloproteinase 1
TNF: Tumor necrosis factor
TNFR: Tumor necrosis factor receptors
TTD: Therapeutic Target Database
VCAM-1: Vascular cell adhesion molecule-1
VEGF: Vascular endothelial growth factor
VEGFR2: Vascular endothelial growth factor receptor 2.
